# Unpacking the Emergency Health Kit of international humanitarian medical aid 1978–90: How humanitarian standards and supply chains became global

**DOI:** 10.1017/S1740022825100223

**Published:** 2025-12-22

**Authors:** Maria Cullen, Bertrand Taithe, Janelle Winters

**Affiliations:** Humanitarian and Conflict Response Institute, University of Manchester, Manchester, UK

**Keywords:** humanitarian medicine, Médecins Sans Frontières, World Health Organization, logistics, standardization, essential drugs lists

## Abstract

This article considers the history of Emergency Health Kits established by United Nations agencies and the larger medical non-governmental organizations of the 1980s to analyse the significance of standardized responses in humanitarian emergencies. We argue that, far from being a rigid and immutable response, the kits reflected a (not universally realized) desire to standardize and control both supplies and medical care from international organizations. As such, humanitarian medical practice remained a disputed field in which each object or drug was negotiated at the risk of creating innovation traps. Coming at a time of increasingly global logistics capacities, the Emergency Health Kits became a central feature of a more coordinated global marketplace of humanitarian aid. The kits’ promise to provide rapid transport of emergency supplies to crisis settings across the world was often experienced as a construct, with long delays and logjams in certain regions. Even so, humanitarian organizations were agents of globalization because they imagined a system of centralized production in the Global North and supply to isolated and/or insecure locations across the world.

## Introduction

Anthropologists of humanitarian aid such as Joël Glasman, Peter Redfield, and Tom Scott-Smith have long noted that the material culture of aid frames what is ‘possible’ and imaginable in the delivery of care.^1^ Redfield depicted the development of aid kits as a marker of difference between humanitarian medicine and development work, and as a codification of what would (and could) be attempted.^2^ Scott-Smith and Kristin Bergtora Sandvik have further argued that the material culture of innovation which developed in earnest as a separate field of industry since the 1980s shaped humanitarianism as a cult of the new (‘neophilia’), engaging with neoliberal market ideology.^3^ A recent United Nations Children’s Fund (UNICEF) advertisement suggests that kits provide reassurance that standardization is not only possible but a core premise of centralized international aid; from acute malnutrition response to education support, ‘so much of it fits in a box’.^4^ Scholars also argue that humanitarian objects can possess a form of agency such as that defined by Bruno Latour, as ‘immutable objects’ retaining their shape and influence on practices in different contexts.^5^ Along those lines, some contemporaries envisioned that kits would standardize the practices of humanitarian medicine. In doing so, they function to a certain extent as a ‘black box’, obscuring the internal complexity of negotiations over practices that the kit embodied.^6^ This history in the global trade of ideas and norms is an example of the material and symbolic investment that international actors imbued objects with to embody a set of reformist values in the late twentieth century.

**Figure 1:**
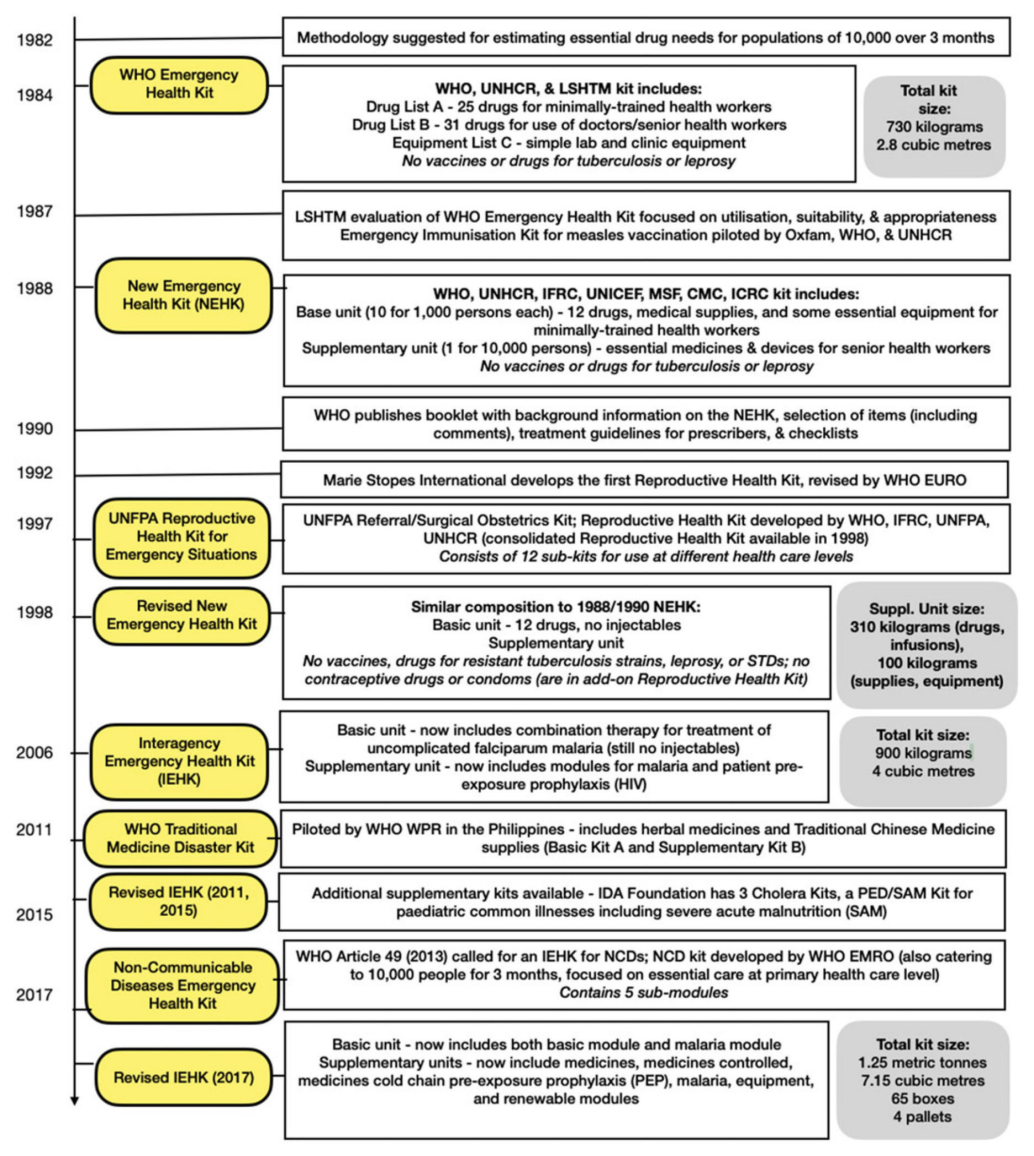
A chronology of Emergency Health Kits, 1982–2017.* Key: WHO = World Health Organization; UNHCR = United Nations High Commissioner for Refugees; LSHTM = London School of Hygiene and Tropical Medicine; IFRC = International Federation of Red Cross and Red Crescent Societies; UNICEF = United Nations Children’s Fund; MSF = Médecins Sans Frontières (France); CMC = Christian Medical Commission of the World Council of Churches; UNFPA = United Nations Population Fund; WPR = WHO Regional Office for the Western Pacific; EURO = WHO Regional Office for Europe; EMRO = WHO Regional Office for the Eastern Mediterranean; NCDs = non-communicable diseases; HIV = human immunodeficiency virus. * Tracking the development of the kit in the 1980s is particularly important because even official WHO and UNICEF publications have incorrectly stated that the first Emergency Health Kit was developed in 1990. (See: UNICEF, *The Interagency Emergency Health Kits: Information Note UNICEF Supply Division* (2025), accessed on 27 November 2025, https://www.unicef.org/supply/media/24426/file/IEHK-2024-Information-Note-2025.pdf; WHO, *The Interagency Emergency Health Kit 2011: Medicines and Medical Devices for 10*,*000 People for Approximately 3 Months* (2011), accessed on 14 November 2025, https://www.who.int/publications/i/item/9789241502115; WHO, *The New Emergency Health Kit 98, 2nd ed*. (1998), accessed on 14 November 2025, https://iris.who.int/handle/10665/42170.) This figure is based on the above references and numerous documents cited elsewhere in the manuscript.

Indeed, it is tempting to ‘over materialize’ the kits. Yet, although they are described in primarily material terms in the first published document on the kit in 1984,^7^ Emergency Health Kits were, and are, most often lists of products deemed essential in the initial phase of a humanitarian programme or emergency, rather than ready-made boxes. Figure 1 shows the evolving iterations of the World Health Organization (WHO)’s Emergency Health Kits from 1982 to the present.

Debates pertaining to the content of the boxes and the items on the lists were at the heart of humanitarian negotiations on standards, and are deeply interwoven with the production of essential drug lists in international health. This article considers the extent to which the Emergency Health Kit, as developed for humanitarian settings in the 1980s, shaped the practices of its users (or vice versa). By delving into the confrontations that animated discussions over the kit in the late 1980s, we unpack the kit’s alleged purpose to reveal the complex norm negotiations that it embodied. We explore the history of the Emergency Health Kit through two broad thematic areas: its influence on people and clinical practices, and on the control of products and markets. We argue that a careful examination of the genealogy of Emergency Health Kits since the 1970s nuances considerably the determinant role that has been attributed to them.

Our analysis is based on primary document analysis from the available established archives of the normative actors involved in conceptualizing and producing the kits, the WHO and United Nations High Commissioner for Refugees (UNHCR), as well as large international non-governmental organizations (INGOs) that played a major role in advising on the kit’s scope or operationalizing it. Médecins Sans Frontières (MSF, Paris, France) incubated and advised on the components of the Emergency Health Kit from its origins in Cambodia. Oxfam (Oxford, United Kingdom) piloted the immunization kits and was consulted on kit contents. Also key was the Christian Medical Commission (Geneva, Switzerland), which played a major role in drug procurement during the 1980s in countries like Somalia and was a sponsor of the second iteration of the kit (the New Emergency Health Kit), while leading major conversations on the role of traditional medicines in emergency settings. We also consulted new archives gathered since 2021 at the Humanitarian Archive in Manchester, particularly the personal papers of former MSF, WHO, and UNICEF staff from the 1970s–1990s, including Jacques Pinel, Rudi Coninx, and Ron Ockwell. Finally, we draw on more than forty oral history interviews that we conducted with humanitarian medical professionals who worked in Somalia, Vietnam, Cambodia, Afghanistan, and Sudan, among other countries, during this period. These interviews will be made available in the near future within the Humanitarian Archive’s oral history project. It is important to note that many archives have disappeared in recent years or that many records are currently closed indefinitely.^8^

Unpacking the Emergency Health Kits allows us to contribute to three broad – and often under-studied – themes in a global approach to the history of humanitarian medicine.

First, the kits are deeply interwoven with the wider history of geopolitics and pharmaceuticalization.^9^ As the first section of this article describes, the kits emerged as a practical response to the logistical challenges of moving health goods in a barely developed humanitarian market, and to the bewildering diversity of medical and pharmaceutical norms among aid providers. They reflected grave doubts UN agencies and voluntary organizations (Volags, later known as non-governmental organizations, NGOs) had over the quality control processes of local drug suppliers, particularly within Sub-Saharan Africa. These issues on the variability of drug quality control later became a major driver of the WHO’s prequalification programme for drugs.^10^ Jeremy Greene has described the ‘plasticity’ of the concept of essential medicines that evolved at the WHO from the mid-1970s,^11^ and Maurice Cassier and Carine Baxerres have explored how the WHO and INGOs negotiated updates of essential drug lists, with the registration of new therapeutic classes (like artemisinin-based combination therapies for malaria and antiretrovirals for HIV/AIDS), and the tensions that arose around local drug production.^12^ The contribution that kits and humanitarian pharmacists made to these debates and to the rationalization of centralized procurement models for medicines^13^ (sometimes described as promoting ‘over-quality’)^14^ at UN agencies are less explored.

Second, the Emergency Health Kit provides insight into the globalization of international claims on medical authority and resultant tensions around epistemic power in humanitarian medicine.^15^ Due to the unique geopolitical and logistical challenges that arose at the border of Thailand from 1979, the practices of humanitarian medicine came under widespread, systematic control for the first time. As Kevin O’Sullivan traced in his analysis of the emergence of humanitarian ‘modernity’ in Bangladesh in the early 1970s, and Bertrand Taithe has argued in his study of the Cambodian refugee camps, international ‘epistemic communities’ emerged in humanitarian medicine during the 1980s around social and technical exchanges focusing on common practices.^16^ Decision-making power varied greatly between refugee camps, ranging from UN bodies and the Red Cross attempting to coordinate aid (e.g. Cambodian camps during the Kampuchean crisis) to the national government proactively coordinating and integrating aid within a primary health care approach (e.g. the Refugee Health Unit in 1980s Somalia).^17^ Clinical guidelines for humanitarian medicine were developed to both control expenditure and ensure cohesion in long-term missions – and these guidelines informed the drugs negotiations for Emergency Health Kits. In this sense, deeper analysis of the Emergency Health Kit’s rationalization and scale-up can contribute to the burgeoning body of literature on the quantification of needs, the ‘agency’ given to tools that humanitarians use in the field,^18^ and debates over the role of national authorities and auxiliary health workers in producing customized drug lists. Indeed, the training and reliability of local medical personnel practicing allopathic medicine often became an object of concern, even though traditional medical practices were recognized and partially integrated to the humanitarian response from the early 1980s,^19^ notably to treat mental health.^20^

Third, the health kits were a product of a commodity chain in the large refugee camps and medical emergencies of the early 1980s. The spatial mobility of goods raised issues of cold-chain capacity, the stability of compounds, and the necessity of standardized processes and norms to achieve cohesion in supply chains.^21^ The Emergency Health Kit’s development chimes with major developments in humanitarian logistics, which themselves sought to align with the rise of ‘just in time’ industrial chains of production (i.e. promoting efficiency by ordering products only when they are needed), the globalization of supply chains, and the associated rethinking of space/time constraints.^22^ Our primary interest is in the kit’s influence on norms (people, practices, and power) and products (market shaping), rather than the wider evolution of humanitarian logistics. As humanitarian actors embraced notions of logistics, they partook in the supply chain revolution which claimed to make the world smaller and more efficient for manufacturers and cheaper for consumers. However, these claims were at this stage more of a construct than a reality, with delivery times for the kits sometimes reaching weeks and months rather than days.^23^ The spatial claims humanitarians could make echoed the quality assurance ones made in industry and, by reasserting the primacy of stringent Global North quality assurance processes, they reinforced Western supremacy in the field of humanitarian aid.^24^ This humanitarian logistical expertise presented tensions with the for-profit commercial world (through a ‘Wal-Mart’ model) by the early 2000s.^25^ In such a way, our analysis of the Emergency Health Kits sheds light on the dissemination and imposition of Western standards and material cultures of aid. These may foster commodity chains that ‘create an interdependence that is less visible but often far more effective than political negotiation at the international level’.^26^

Based on these reflections, this article explores the nuanced history of the Emergency Health Kit through its origins and two broad thematic areas: first, the control of people and clinical practices and second, the control of products and markets. These have sometimes been presented as ruptures with previous practice, particularly the Sphere project’s framing of accountability from 1997 in terms of quantified basic needs of displaced peoples.^27^ While the kit has been ultimately subsumed within this ‘accountability’ discourse, our analysis shows that increasing accountability, which would imply openness to criticism and collaboration with local actors, was never its primary intent. Its immediate goal was to assert external controls over resources and quality, and to re-establish trust in medical expertise and authority in humanitarian providers despite chaotic contexts. The kit provided a list of drugs and equipment classified as essential for emergency responses and defined care protocols based on these materials. In so doing, the kit set boundaries around the conduct of medical professionals and health workers, reducing flexibility and the ability to curate care according to specific community or patient needs in emergencies. Thus, it imagined and responded to the ‘emergency’ as a temporal and spatial alternate reality, where only a new and strictly limited vision of ‘humanitarian’ medicine should be practiced by a specific set of professionals.^28^ In this sense, the kit ended up being a not so immutable object but a discursive field or indeed ‘open container’ in which key debates of humanitarian medicine and pharmacy could be focalized.^29^

### Origins and content of the kit

Medical emergency kits can be traced back to military standardized supplies deployed in the late nineteenth and early twentieth centuries.^30^ As an expression of the expertise of humanitarian organizations, and as the physical embodiment of a baseline for action, their earliest manifestation seems to date from the 1970s when Oxfam developed water and sanitation kits (e.g. the Oxfam DelAgua Water Testing Kit of 1974) and nutrition kits for nutrition centres.^31^ There had also been missionary dispensary kits developed by organizations like ECHO (the UK-based ‘Equipment for Charity Hospitals Overseas’ and British purveyor of drugs for the missionary medical market) in the 1960s and the 1970s.^32^

Additionally, emergency medical kits drew from the mid-1970s WHO initiative to normalize the nomenclature of drugs and rationalize their use, which led to the release of a list of 186 essential drugs in 1977.^33^ The list was adapted into a shopping list of first response and essential medicines required to meet the demands of refugees in a temporary camp.^34^ In 1982, the WHO created the first Emergency Health Kit, which included drugs and medical equipment needed by humanitarian organizations to provide for the health-care needs of 10,000 displaced people for the initial three months of a settlement. MSF became a leading organization in establishing specialized medical kits and developed a set of clinical and pharmaceutical guidelines, which it described as ‘a notebook of pathologies with their fundamental symptoms and most likely diagnostics and treatments, lists of standardised drugs, with the dosages and protocols most adapted to the limited means of [medical] practice in poor contexts; [along with] some propositions to organise public health programmes, prevention and health education’.^35^ This manual and its associated drug lists were attempts at shaping a consensus around a unitary (rather than proprietary) kit for emergency humanitarian medicine. These efforts, in turn, reflected the global ambitions of the rapidly professionalizing humanitarian sector in this decade. While this brief chronology seems to establish a neat progress narrative one should be sensitive to the negotiations and contestations that occurred along the way, for the kits embodied social and cultural negotiations as well as aspirations to more consistently effective medical care.

Refugee camps managed by international organizations loom large in the history of the kits. Following the invasion of Cambodia by the Vietnamese army and the overthrow of the Khmer Rouge, the first 30,000 Cambodian refugees arrived at the Thai–Cambodian [Kampuchea] border in late October 1979, launching a mass displacement crisis that would endure for the next decade.^36^ The scale, and later the protracted nature, of the Kampuchea crisis demanded a professionalized system of logistics to supply the network of camps along the border.^37^ As then MSF doctor Vincent Fauveau remembers:

We were under ICRC [International Committee of the Red Cross] coordination which was very effective. We built … They built in one week a bamboo hospital of one thousand beds, with a paediatric and general medicine service. A hospital of one thousand beds built in practically one week by the Thais with Red Cross funding.^38^

**Figure 2:**
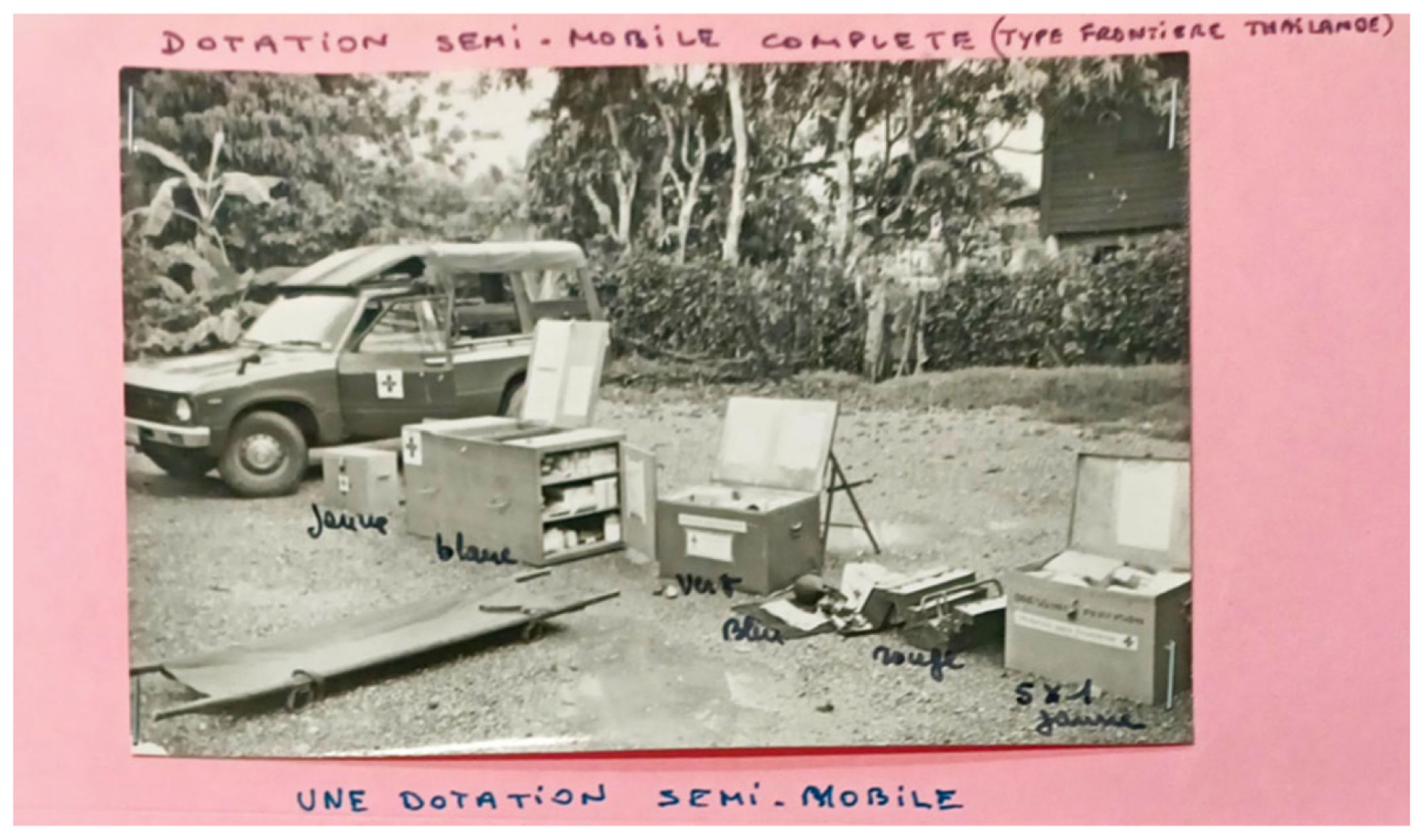
Une ‘dotation semi-mobile’, Papiers Jacques Pinel, Listes, MSF archives, Paris.

This scaling-up could not be attributed to one actor alone but was instead the product of a coalition, coordinated on a voluntary basis in a much more structured manner than in previous interventions. Within weeks, this camp (Khao I Dang) became the largest UNHCR-run refugee camp. Meanwhile, those who failed to cross before the Thai government restricted access were eventually catered to by the separate UN Border Relief Operation (UNBRO). UNBRO border camps required a mobile pharmacy system capable of equipping Volags/NGOs with the means of a primary health care service in the camp’s clinics. Medical organizations, including MSF, entered procurement deals with Thai pharmacists in nearby towns like Aranyaprathet. In addition, MSF improvised *dotations semi-mobiles* (semi-mobile provisions), boxes of ‘essential’ medical equipment and drugs needed to treat trauma injuries which could be driven to the border in trucks at short notice (see Figure 2).

There were several reasons why the Cambodian camps became an incubator for discussions about global norms in the headquarters of WHO and other UN agencies in Geneva. For one, the stability of an internationally run refugee health care programme at the Thai–Cambodian border provided the perfect context for the ‘facilitation of knowledge production’.^39^ MSF had an unbroken presence as a core medical provider at the Thai–Cambodian border from 1979 to the break-up of most of the camps in 1991,^40^ which enabled it and other NGOs to conduct long-term medical research with control groups on topics like Post-Traumatic Stress Disorder.^41^ Also, strong relationships between long-term NGO staff operating in the camps and the WHO provided opportunities for cross-pollination of kit discussions. An increasingly close relationship developed between MSF pharmacist Jacques Pinel and officials in the WHO’s Action Programme on Essential Drugs (DAP), for instance, leading DAP consultant Soren C. Sorensen to travel to Thailand in 1987 to conduct a comprehensive report on drug use in the camps as part of the process of kit revision.^42^ This stands in contrast to standardization efforts that proceeded simultaneously in Somalia. In Somalia, a conscious national ideology of primary health care (influenced by the fact that many Ogaden refugees were ethnic Somalis) placed an emphasis on Somali rather than global standards, and fostered a handover of camp management from international to Somali counterparts (with MSF initially leaving the country in 1983).^43^ As such, there was little sense of *international* ownership of this programme. This may explain why a figure like Pinel emerged from the experiences in Thailand as a significant shaper of global norms, while the pharmacist Beverley Snell and her Somali superiors (Regional Medical Advisor Abdilahi Hassan Farah and Primary Health Care Training Officer Yusuf Mohamed Abdilahi) corresponded only briefly with Sorensen on the kit project.^44^

The complexities of the Cambodian border response conditioned how international organizations professionalized their logistics in the absence of a strong state setting the agenda (as in Somalia). This was a proxy Cold War conflict that captured the attention of Western donors, leading NGOs to grow at a rapid pace. Because NGOs like MSF received funding from intergovernmental bodies such as the European Economic Commission (EEC) and UNBRO in Thailand, they had to produce detailed reports, accounting for how they spent their money.^45^ However, this was also a context where aid diversion by armed groups routinely occurred, adding to the pressure NGOs were under to demonstrate that they could exert control over their resources. They rationalized pharmaceutical management systems accordingly and created clinical guides which doctors and refugee health workers were supposed to follow when prescribing medications.^46^ This impetus to standardize materials and practices gathered pace both on the ground in emergency settings and in the headquarters of UN agencies and NGOs.

In essence, the kit sought to extend this standardization impetus to emergency humanitarian response worldwide. Yet, it responded to a narrow set of health care needs. Despite its name, the kit was not destined to be used for the acute phase of emergencies, such as epidemics, wars, earthquakes, or floods,^47^ or to provide long-term treatment of chronic diseases.^48^ It did not deliver responses to vaccination needs, famines, or endemic diseases like tuberculosis, leprosy, and Human African trypanosomiasis (sleeping sickness). Instead, the kit was intended to provide a normative response for the immediate needs of a displaced population whose access to health care had been severely disrupted.^49^ In an MSF official’s view, this amounted to the provision of essential care to a standard population in a relatively normal state of health in a tropical region.^50^ As such, the kit focused on what was needed to control infectious diseases like cholera and malaria, and to limit maternal and child health issues. The drugs in the kit provided for both refugee health workers and doctors or nurses, restricting the drugs that required the most medical supervision to the latter. In the 1987 draft of the ‘basic’ health worker list, the contents destined to their practice included drugs for pain management (paracetamol), ointment for eye infections (tetracycline), antibiotics, and nutrition and maternal care (ferrous sulphate, folic acid). The ‘supplementary’ kit for health professionals included anaesthetics (ketamine, lidocaine), stronger analgesics, antiepileptics, anti-infectives, cardiovascular drugs, disinfectants, and psychotherapeutics. All injectable drugs and intravenous treatment materials were reserved for the ‘supplementary’ kit.^51^ The quantities envisaged in the kit were understood as estimations, which may not meet the disease prevalence rates observed in all types and scales of emergency. While in one crisis the drugs in the kit could last for six months, in another they could run out in one month.^52^

Despite the intentionally narrow remit, the task of creating a standardized kit was an ambitious one, and as such it was ridden with tensions. In particular, the specific drugs included (and excluded) and their quantities were contested by many officials as inappropriate for clinical and logistical reasons, and the initial splitting up of the kit into three subsections (drugs for doctors, drugs for refugee health workers, and equipment) was seen to be overcomplicated and counterproductive. In 1986, the WHO and a constellation of other actors including UNHCR, UNICEF, MSF, the League of Red Cross and Red Crescent Societies, ICRC, the CMC, and others embarked on a full-scale evaluation and revision of the kit contents.^53^ Of these actors, UNHCR and MSF played prominent roles, with UNHCR scrutinizing procurement and testing in the field, and MSF providing the therapeutic guides that accompanied the kit and codified their use.^54^ The proposal for this project emphasized that, ‘A rational use of drugs requires quantification of needs, training of health workers, and an effective supervisory system’, indicating that these had been barriers to the roll-out of the kit in the past.^55^ Its stated goals were to include lists, treatment guidelines, packing and storage instructions, and suggestions for coordinated activities. The revised kit retained the separation of components according to the training level of those handling it but contained only two subsections: medicines and equipment for doctors, and medicines and equipment for health workers.

In the end, the input beyond the UN system became so crucial to the reformulation of the kit that it was not named the WHO-UNHCR Emergency Health Kit as intended, but interchangeably labelled the ‘new’ or ‘revised’ Emergency Health Kit (see Figure 1).^56^ The revised kit was officially launched in 1990, yet the disagreements raised in the revision process over the contents of the kit (and sometimes the very viability of the kit concept itself) endured. By 1992, MSF had introduced up to fifty ‘supplementary’ kits in the field^57^ to address specific unmet needs.^58^ Indeed, the many supplementary kits now produced by the WHO to cater to more specialized emergency requirements (such as trauma and emergency surgery, cholera, non-communicable diseases, cold-chain medicines)^59^ are ‘frequently reviewed and updated to adapt to changing needs based on experience in emergency situations’.^60^ In the late 2000s, the WHO Regional Office for the Western Pacific even piloted a basic and supplementary traditional medicines Emergency Health Kit.^61^ This suggests that the kit has become less a standardized object of humanitarian medicine and more a discursive field of action, constantly shifting yet nevertheless perennially at the centre of debates about what an ideal programme of universal, emergency humanitarian medicine should look like.

### Control of people

The kit responded to the diversity of health providers that existed in emergency settings by formally distinguishing the drugs they were permitted to handle. According to WHO official Hans Hogerzeil, the basic units to be used by refugee health workers were for ‘consultations at the most peripheral health care level’, while the supplementary units provided for ‘outpatient consultations at the first referral level’.^62^ This need and the demand from coordinators in the field to control medical practices was viewed as a rational response to an excessive freedom hitherto enjoyed by refugee health workers and expat medical professionals. This was clearly outlined by MSF pharmacist Jacques Pinel, who stated in 1986 in a letter to the WHO’s Soren C. Sorensen that at MSF, ‘We wholeheartedly believe in the utility of such a kit, not only for managing the emergency care of a displaced population, but also because of its incidental effects’, including the ‘standardisation of “rotating” expat personnel’s conduct’.^63^ The kit was therefore intended to control the practices of health care providers at all levels. However, this was more intellectually and materially consequential for refugee health workers because the kit in effect formalized a hierarchical distinction between international and national staff. This crystallized an already established understanding of who possessed ‘expertise’ (and who did not) in aid work, reminding us that colonial continuities were baked into the professionalization of a global humanitarian sector in the late twentieth century.^64^

The desire to standardize medical practices was not ‘incidental’ as Pinel remarked, but a driving force of the kit project. The kit institutionalized existing trends within specialized UN agencies (UNHCR, UNICEF) and missions (UNBRO), and several NGOs, including Oxfam and MSF. Take, for example, MSF’s involvement in Thailand from the early 1980s. Vincent Fauveau was the first Head of Mission appointed for MSF’s expanded emergency programme in 1979.^65^ While managing the fast-growing team, Fauveau realized that new (expatriate) volunteers rarely had ‘any idea of the specificities of humanitarian and emergency medicine which they had to practice’.^66^ Bioforce (a humanitarian logistics training platform for mostly European aid workers), which signalled the beginnings of a professional culture of humanitarian action, was not set up for another four years.^67^ To address this issue, Fauveau created guides which told medical workers ‘what they had to do when they were on night duty, when they encountered cases of malaria, severe measles with complications, severe diarrhoea, dysentery, tetanus etc’.^68^ Other organizations responded along similar lines. Around 1983, UNICEF published a first draft of its Emergency Manual^69^ and in 1984 Oxfam published its guidelines on selective feeding, which were initially written as an internal document during the Ethiopian famine of 1974.^70^ The production of guidelines and manuals went in parallel with kits and completed a process of standard setting, work expectations, and quality thresholds common to all organizations at the time. These processes, based on discipline and consistency, did not invite contestation or debate and they often kept the compromises they embodied unseen and unspoken. The lack of transparency made these guidelines and manuals representative of biopower in the Foucaultian sense.^71^

The impetus to control and discipline practice was even stronger regarding locally recruited health workers in the ‘Third World’. Indeed, issues of (lack of) trust permeated virtually all relationships between expatriate health professionals and local colleagues at the time, especially with ‘medics’ (often summarily trained health workers and themselves members of displaced populations) in emergency settings.^72^ Tellingly, in drafting the UNICEF Field Manual, Ron Ockwell stipulated that staff should ‘determine whether there are any individuals available in the country with appropriate qualifications and personal qualities to undertake the particular “professional” functions required’.^73^ This seemingly well-intentioned policy highlights that this approach was an exception to general trends – the fact that international workers were encouraged to investigate whether ‘any’ individuals existed in-country with relevant skills demonstrates the conceptual distinction drawn between Western medicine as a realm of self-governed ‘professionals’ and the Global South, where such a designation was not as reliable a norm. Historians have noted how humanitarians and development workers often failed to take account of the structural effects of colonialism on chronically poor and disaster-struck societies.^74^ In the context of persistent global inequality that humanitarian action operated in during the 1980s (and continues to operate in today), what were presented as apolitical professional guidelines often served to reinforce racialized assumptions about the ‘Third World’.

This begs the question – did humanitarians recognize other expertise? In the Cambodian refugee camps, some provision was made for traditional Khru Khmer practitioners,^75^ but this was always with the caveat that supervision by Western professionals was needed. Indeed, in 1988 (almost a decade into the Cambodian refugee crisis and a year before most of the camps were dismantled and refugees repatriated), UNBRO officials spoke with a sense of novelty of the need to aim for ‘Khmer self-management’ in the future of the relief programme, which had hitherto been largely ‘vertical’ in service delivery style.^76^ Instead of relationships between ‘professionals’, the majority of operational interactions between expatriates and local health workers were between doctors or nurses and the aforementioned refugee medics (also commonly referred to as refugee health workers). Here too, a tension existed between the desire to foster local ownership of humanitarian relief, influenced by the rise of primary health care discourse at the WHO in the late 1970s,^77^ and the general trend towards more technical, quantifiable policy interventions in the Global South in the 1980s, which were much less accommodating of flexibility of care and allowance for the autonomy of local health workers.^78^ For example, the first draft of UNICEF’s manual in 1983 repeatedly declared that health services should be developed ‘with’ rather than ‘for’ refugees, and an emphasis was placed on preventive rather than curative care, with services ‘based on the concept of primary health care’.^79^ This even extended to a professed commitment to take account ‘of the experiences of traditional healers and midwives’, yet, crucially, this was contingent on ‘supervision and referral’ to professionals being available. An underlying paternalism governed cooperation between international professionals and local health workers in the field, whose knowledge was theoretically valued yet never truly seen as an alternative to the expertise of Western medical professionals.^80^ There was also the issue of what level of medical care the refugee health workers should be entrusted with. Injectable medications were particularly contentious; in the early 1980s, basic health workers in many countries (including in Africa) had been ‘provided with [injectable] procain-penicillin because of patient compliance (a shot a day takes the illness away)’ but concerns over sanitation and safety (not least because of the ‘current situation with regard to AIDS’) led all injectables to be removed from the kit for basic health workers.^81^ Medical ethics and professional standards of care, albeit associated with ‘appropriate technologies, defined principally in terms of maximizing efficacy and minimising costs’, had to be upheld against ‘misconstrued’ primary health care.^82^ To borrow from Scott-Smith’s work on the relief of hunger, the kit can also be seen as a ‘low modernist’ device in that it combined the utopianism of high modernist ideals (faith in science and universal rationality) with ‘a more pragmatic and business-friendly’ understanding of what was possible in low-resource contexts.^83^

The way the tensions created by this form of standardization manifested in the Cambodian refugee camps in Thailand is revealing. To gather data on health care systems in long-term displacement settings, Sorensen visited the Cambodian camps in 1987. As the camps no longer represented acute crisis settings, Sorensen expected to find a low consumption of drugs, yet instead discovered over-consumption. Sorensen remarked that ‘diseases treated by various levels of health workers were not always clearly defined and practices differed between camps’. In addition, he concluded that:

the high consumption of drugs was not found to be explained by high morbidity, but rather by poor prescription practices. The pressure on medics from fellow refugees to prescribe drugs was considered a major problem in attempts to enforce a rational use of drugs. The lack of constraints on requests for drugs was also found to be a major problem in attempting to enforce a more rational drug policy.^84^

In the 1986 project proposal for the kit’s revision, it was acknowledged that UNBRO faced challenges in introducing ‘standard treatment schedules for Khmer medics’. The proposal also made the alarming claim that less than 25 per cent of patients treated in UNBRO camps actually required curative treatment with drugs, and raised the possibility of including vitamins as a placebo drug, suggesting a distrust in not only the medics’ prescription tendencies but also the patients themselves.^85^

Anxieties over aid diversion by warrior refugees were central in this context.^86^ Indeed, in the years preceding Sorensen’s visit, MSF had encountered resistance when it attempted to assert control over medical resources in Dong Ruk, a camp run by the anti-communist Khmer Serei faction.^87^ Khmer Serei leaders counted on access to medical materials to supply nearby military satellite camps, and when MSF responded to UNBRO calls for reduced expenditure^88^ by cutting the quantity of drugs and materials entering the camp, refugee health workers went on strike.^89^ After MSF agreed to increase the quantities of some medicines in the night-duty box, the strike ended. This small concession illustrates the inability of external actors to fully control the use of medical materials in the Cambodian camps.^90^ In December 1984, the security situation (with Vietnamese attacks on camps) had become so dangerous that access to the camp for foreigners was difficult and ‘boxes with medicines enough to make the hospital function for 3 days [were] left in the camp’, once again underlining the challenges of enforcing control of drugs in this setting.^91^ In 1986, MSF officials continued to grapple with this issue as they discussed what data should be used as the basis for deciding the drug quantities to go into the kit. Officials requested the collection of information from the field on the distribution of pathologies, which would be sent to Paris for epidemiological calculations of average pathologies. By comparing expected drug consumption based on this disease distribution with real consumption levels, they could attempt to catch ‘poor application of protocols, theft and other loss of stock’.^92^ The diversion of the materials of aid by military actors in Cold War conflicts should not be forgotten as the context in which such distrustful relationships developed between medical actors in humanitarian settings.

This is not to say that NGO management officials did not also sometimes face stiff resistance when attempting to standardize the conduct of international medical professionals. Jean Rigal, MSF’s medical director in the 1980s, recalls how an expatriate paediatrician operating in Sri Lanka once refused to use the antibiotic listed in MSF’s clinical guide for the treatment of a severe illness he encountered in a child there, and instead went to the local pharmacy to purchase a different antibiotic he believed would be more effective.^93^ Similarly, Martijn Blansjaar, a field logistician who later became head of logistics at MSF Netherlands, remembers the tensions caused by using the Emergency Health Kit in the field, which necessitated ‘a whole education of the medical staff … because in order for us to deliver assistance of a standard quality in those environments, supply chain was essential … and the only way we could make that happen was by limiting the amount of choice’. In ‘working with doctors who’ve never thought about supply chain at all’, this sometimes led to misunderstandings and doctors asking ‘“why haven’t you got x, y, z drugs for me?” and being very irritated when they weren’t available’.^94^ As these examples demonstrate, the kit was intended to control people and their ability to use professional judgement in medical care at all levels, including international staff, but it was also intimately linked to broader issues and debates pertaining to products, market forces, and scientific controls.

### Control of products and markets

The debates on the procurement of drugs revolved around some very practical considerations. The first was to identify the name under which different products were commercialized. The use and posology (dosage patterns) of drugs varied in different medical cultures. Teams originating from Germany wished to use products that other teams had limited experience with, while French drugs contained only half the active ingredients of their American counterparts trading under the same name.^95^ At a fundamental level the diversity of medical and pharmaceutical practices was most salient when fast turnover of volunteers brought these cultures into the same space. This was particularly the case for teams of medical INGOs which recruited broadly; MSF, for instance, underwent rapid internationalization in the 1980s with new branches appearing in Europe (Belgium, Luxemburg, Netherlands, Switzerland, Spain, and Greece).^96^

Jacques Pinel focused on engaging with the WHO essential drugs lists.^97^ Renewed momentum to establish a common list of drugs named scientifically rather than through the brands of their developers was already under way by the mid-1970s but accelerated in the context of international humanitarian responses.^98^ The list which became the basis of the emergency kits was composed of two complementary drug lists and excluded vaccines and drugs for the control of certain communicable diseases, justified because, ‘1. Many countries have national disease control programmes and 2. It is necessary therefore, first to investigate the incidence of these diseases among the refugees and then discuss the problem’.^99^ Beyond respecting the epidemiological sovereignty of states, the kit made several assumptions: that half of a displaced population would be below the age of fourteen and that a referral process could take care of patients requiring ‘more sophisticated treatment’. According to MSF’s estimation of the needs of people in an emergency setting, the expectation was that 500 might be ill at any given time.^100^ An MSF report on the kit revision in December 1986 recommended that the list of drugs in the kit should be as reduced as possible, and that reductions should avoid any duplications of molecules (it was ‘not useful for example to have both Chloaramphenicol and Promethazine’), and also be tailored according to form (avoid syrups and drops) in accordance with international dosage variations.^101^

Weight and cubic meterage became key characteristics of the discussion of products and of what producers could provide. The 500 kg basic equipment kit for the Rural Health Centre devised by the British supplier ECHO had been set up from a development perspective and its stock catalogue clearly targeted supplies for very basic dispensaries and primary health care structures for a cost of £5,000 in the 1980s.^102^ ECHO essentially offered supplies for the set-up of a Rural Health Centre as the nexus of a web of Village Clinics. Its routine kits did not meet the acute needs of displaced people fleeing wars but instead corresponded to the supplies required by dispensaries in the bush, based on the logic that ‘most authorities concerned with the development of health services in under-developed countries agree that the emphasis must be away from the high-cost traditional Hospital towards the establishment of Rural Health Centres’.^103^ The ECHO kit contained basic drugs, medical equipment, and consumable supplies, ranging from 300 tongue depressors to four batteries for a Penlight torch. A Village Clinic, designed to be subordinated to a Rural Health Centre, would be further pared down in medical provisions and diagnostic tools. In comparison, the kits developed for MSF Belgium following the WHO classification of essential drugs were structured to supply three levels of care: a dispensary without a prescribing doctor, a medically staffed health centre, and a specialized centre. The full vertical structure including equipment represented twelve parcels and 2 tons of weight.

Pinel compared UNHCR and MSF kits line by line and noted what choices had been made for inclusion in the boxes.^104^ Our analysis of the archives show that these very practical questions involved complex negotiations and diffuse arbitration by self-appointed expert committees.^105^ This scientific epistemic community emerged from a field of practice and on-the-ground testing rather than from laboratory science. In turn, newly emerging commercial suppliers and those evolving from missionary medical activities (such as ECHO) were challenged on the quality of their offerings. Packaging had to be heat resistant, and labels had to stick in tropical conditions.^106^ The provider organization for ECHO, Missionpharma, was thus challenged repeatedly in the 1980s on the suitability of its products.

As the Emergency Health Kits were designed to meet basic pharmaceutical and surgical needs, they were normally conditioned for this purpose only. Yet, because they were by nature focused on meeting the expected needs of 10,000 as part of a baseline response for a period of three months, they also served to establish the core of a local pharmacy and the end point of a supply chain. The basic unit of each kit was questionable in terms of its versatility and suitability for smaller emergencies. In December 1986, the WHO’s Hans Hogerzeil commented that the idea of breaking the kit down into 25 kg parcels for 1,000 people for a month was ‘tricky’; it had obvious benefits, being ‘easy to handle, and easier to supply exactly according to expected needs’ compared to the kit for 10,000 people which ‘was much too bulky, and expensive too’. However, the downside was that ‘to put all different items in one (small) kit of 25 kg may reduce their quantities to unpractical and expensive sizes’. He suggested that a kit for 3,000 with additional supplies could make more sense ‘as the range of drugs will be greater, and quantities probably smaller’.^107^

More fundamental were the debates on quality and posology. The medical culture of old colonial empires directly influenced the language and uses of drugs by NGOs. Local choices of drugs and practices correlated to NGO resources (for instance the use of Ketamine as an anaesthetic) and reflected best adequation with training and availability. The rationale for a universal Emergency Health Kit was thus not assured. While explicitly presented as a compromise of best available scientific evidence and cost effectiveness, critics challenged kits as decisions taken from afar in ignorance of local customs and specific needs. The world of humanitarian medical procurement proved to be intractably cultural even when humanitarians sought to establish it as a universal.

Emergency Health Kits epitomized supply-led humanitarian response. They also entailed industrial-scale processes. The financing of the products entering the kits – including gifts from the pharmaceutical and medical industries – was never entirely stable. The warehousing of the kits was set up in Denmark for UNICEF^108^ and Bordeaux for the French section of MSF in 1986,^109^ while the Dutch followed International Dispensary Association (IDA) advice, but the kits could be assembled from lists anywhere where quality could be assured. The supply of goods across the world engaged with the development of airfreight in the 1980s as well as the considerable growth of naval freight. Warehousing the ingredients of kits in preparation for a humanitarian emergency entailed accounting notions of depreciation and inventory write-offs as well as the technical demands of real-time stockkeeping.^110^ Some of the fiduciary responsibilities of humanitarianism took a very concrete form through immobilized capital kept either in store or prepositioned^111^ in the expectation of demand.^112^ The demands for extremely rapid procurement thus depended on logistical constraints for which humanitarians were initially not equipped.^113^ Only Oxfam and UN agencies had developed any kind of know-how in this respect prior to the 1980s. UNHCR and UNICEF themselves relied on regionally operating supplier and procurement actors in most of their previous operations.

The desire to control procurement and supply revealed concerns over consistency and quality assurance in a booming marketplace. By 1977, worldwide *production* of pharmaceuticals was $64.52 billion annually, with the share of developing countries only 11.43 per cent of this – and Africa just 0.56 per cent. Yet, in the period 1968–1973, developing countries more than doubled their drug *imports* (with an average annual growth rate in imports of 15–17 per cent by 1977). Within international health more widely, the transnational pharmaceutical network was booming, and outpaced the capacity of national regulatory agencies in these often recently decolonized countries.^114^ European providers were increasingly operating in one consistent pharmaceutical market regulated by national and European agencies, matching the US federal and Japanese drugs agency quality standards,^115^ which would harmonize officially in 1989.^116^ In contrast, the rest of the world presented a much more complex marketplace with local ecosystems of drug production, homologation, and often ill-controlled supply chains. WHO officials based in Geneva and their UN correspondents were familiar with European and American quality controls and set them as ‘stringent’ examples of the ‘gold standard’ which justified centralized procurement. The WHO’s prequalification programme, which initially launched to assist UNICEF’s selection of high-quality vaccines in 1987, was only expanded to cover medicines in 2001.^117^ The WHO Prequalification of Medicines programme (initially focused exclusively on drugs for HIV/AIDS, tuberculosis, and malaria) was largely a response to rising concerns in the 1990s over the procurement of generic drugs and novel HIV/AIDS fixed-dose combination therapy antiretrovirals, as well as paediatric formulations, which were produced under relatively lax Indian regulations.^118^ By 2001, when only one-third of national drug regulatory authorities were deemed of high enough rigour,^119^ the WHO largely relied on UNICEF, IDA, and MSF quality-assurance mechanisms rather than national capacities.^120^ By then humanitarian players and UN agencies had established their credentials through rigorous drug quality testing, selection processes, and clinical engagement with transnational pharmaceutical companies (such as through MSF’s epidemiology satellite Epicentre, launched in 1986, and the inter-agency Special Programme for Research and Training in Tropical Diseases, TDR).

This considerable achievement should not obscure the contests along the way. Beyond the critique of supply-led humanitarianism, which applied to all humanitarian workers and programmes, there were some serious challenges to the bottlenecks that centralized supply chains produced. Purchasing from authorized suppliers and engaging long-distance supply chains created frictions at the time of the order, gathering of supplies, clearing of the shipments (in particular for psychoactive drugs),^121^ and clearing on arrival and through customs. Delays (up to nine months in Somalia)^122^ and risks accrued by distance echoed imperial commercial networks which had tied so many countries to their colonial markets. In a visit to the WHO Regional Office for South-East Asia in New Delhi, a Drug Action Programme official found that a trial of ten kits had not been received well in 1987, with wide-ranging complaints on the ‘content, bulkiness, difficulties in redistributing its content and price of shipping from distribution points in Europe’.^123^ Besides issues of accordance, the kit supply chains could be wasteful in acute but small emergencies. WHO officials in the Western Pacific regional office were thus highly sceptical of a supply chain originating on the other side of world. Ultimately these tensions around the kits reflected the tensions around the consolidation of WHO normative power in Geneva.^124^

The control of markets by large INGOs and UN agencies was criticized for its monopolistic logic prior to the 1990s debates on access to anti-retroviral drugs, and sharpened in cases when supply chains failed to deliver the kit in a timely basis. INGOs claimed to encounter several difficulties in purchasing kits from UNIPAC (UNICEF’s Copenhagen-based freighting company), when the supply of kits advertised to be shippable ‘within 48 hours’ in fact took between fourteen and thirty days.^125^ The bureaucracy of large warehouses could cause major delays and the UNICEF annual stocktake clashed with emergency needs in Mozambique, for instance. With European inflation in the late 1980s, there were also vacillations in kit pricing and variations in contents according to the stock available in the UNIPAC warehouse.^126^ The alternative was, of course, recourse to a free marketplace. Ideologically, one could imagine the 1980s to have been an ideal time for faith in the market to trump other considerations.^127^ Yet the medical marketplace is not like others and the risks entailed in open market procurement in terms of vital outcomes and reputational damage were such that the kit could take hold,^128^ with a number of caveats.

The Emergency Health Kit itself soon became a more composite aggregation of health kits rather than the unitary model arising from a single basic essential drug list of the early 1980s. These kits built on empirical evidence and on the analysis of recent programmes rather than any simulation of future needs. Meanwhile, the pre-deployment of some kits in regions prone to disasters was attempted, and indeed recommended in the 1987 draft of the Emergency Health Kit, but often abandoned for logistical reasons of secure warehousing in unstable environments and issues with drug expiration.^129^ In this sense, the kit (and guides that accompanied it) conjured an exclusionary zone of medical practice. This was made explicit in the narrow, yet contradictory conceptual boundaries set by the kit around the ‘emergency’ it was intended to respond to. Perhaps more of a historical construct than a reality, the kit never truly fixed practices for long or created an entirely uncritical and obedient culture of medical practice in the field. However, in the long term, it did implicitly establish an accepted domain of action for humanitarian medicine.

One area in which this was the case was for antibiotics. In the revised version of the kit, only one antibiotic (Cotrimoxazole, ‘also the drug of choice for respiratory infections’) was to be included.^130^ Sorensen remarked to a Danish pharmacist in 1986 that ‘this is indeed a major step, which may not find full support among specialists to be consulted, and we may have to provide a limited amount of [injectable] procain-penicillin for children who vomit or who otherwise may not be able to take the drug by mouth’.^131^ One of the advantages of procain-penicillin injections was that they were administered daily; Sorensen thus wondered if a daily dose schedule for Cotrimozaxole could be designed by his colleague.^132^ This decision to choose just one broad-spectrum antibiotic for use in emergency situations was not necessarily supported by other pharmacists and health professionals influenced by the Essential Drugs Programme in humanitarian settings. In Somalia, for instance, the primary health care–inspired Refugee Health Unit provided for the use of more than one antibiotic in its list for community health workers, which was otherwise ‘fairly similar’ to the list for basic health workers in the kit.^133^ In the era of the kit, debates soon revolved around *which* antibiotic would be included as essential, rather than *how many* antibiotics to bring to an emergency. In this sense, the kit can fruitfully be compared to other humanitarian ‘tools’ like the mid-upper arm circumferance (MUAC) tape and Plumpy’Nut because, in influencing the types of questions asked and limiting the boundaries of imagination, it functioned to some extent as a black box of emergency medicine. Given the increasing threat presented by antimicrobial resistance since the 1980s, it is worth highlighting the kit’s role in limiting discussions on antibiotics in this way. According to the WHO, there were an estimated 1.27 million deaths in 2019 attributable to antibiotic-resistant bacteria, yet research and development of new antibiotics remains slow due to lack of investment.^134^ Antimicrobial resistance is much higher in low-income countries, where broad-spectrum antibiotics are often used for longer periods than in wealthy nations, and conflict situations in particular are recognized as ‘major incubators’ for antimicrobial resistance.^135^ In choosing to include only one broad-spectrum antibiotic, the kit can plausibly be correlated with a broader global medical culture which perpetuated the over-use of specific antibiotics in the Global South, where antimicrobial resistance now presents the greatest threat.

The capacity for the kit (and the system of standards it represented) to act as a brake on innovation, by making its workings closed to contestation or challenge, was also visible in the case of the anti-malarial drug chloroquine. Difficulties were initially encountered in creating universal standards for the use of this drug due to safety concerns around different dosage levels. In Francophone countries, the practice was to vary dosage levels between 100 and 150 mg tablets, yet in Anglophone settings (and in the WHO List of Essential Drugs), only 150 mg tablets were used.^136^ In designing the kit, logistics won out over safety concerns – only 150 mg tablets were included and the drug was approved for a wide use, including in the treatment of all child fevers, despite some discomfort among MSF officials on this point.^137^ There was also growing awareness throughout the 1980s of chloroquine resistance becoming a problem. Indeed, discussions about removing chloroquine from the kit in the late 1990s and early 2000s reflected a sense of urgency but also an awareness of inertia – after circulating the 1988 draft, an official remembered, the Division of Control of Tropical Diseases/ Malaria (CTD/MAL) was requested to comment on the kit and communicated ‘the inadequacy of maintaining chloroquine as a unique antimalarial treatment in these kits’.^138^ An over-reliance on chloroquine thus represented a ticking time-bomb and epitomized the collective rather than clinical patient care model envisaged by the kit. Cost concerns (and political apprehension in the west) prevented the wide uptake of a viable alternative drug derived from traditional Chinese medicine (artemisinin and artemisinin-based combination therapies), and recommendations for anti-malarial drugs now emphasize the importance of tailoring drug choice to knowledge of local resistance patterns. In this sense, the idea of an emergency kit fit for use anywhere in the world has been frustrated by the complexities of antimicrobial stewardship in recent decades.^139^ When difficult compromises between cost and scientific recommendations are embodied in the form of an international kit, unpacking the kit becomes contentious and threatens to unravel hard-won agreements, if not scientific consensus.

## Conclusion

To return to the lists of drugs and contents of the kit: what kind of ‘humanitarian medicine’ were they bringing into being? In 1987, WHO official Sorensen commented that his colleague Hans Hogerzeil’s approach to humanitarian medicine was simply ‘far too sophisticated for an emergency health kit’ because it was ‘based on diagnosis and not on symptoms’.^140^ This shows how, through internal debates about the kit, WHO officials and their peers in NGOs were negotiating the boundaries of the underlying logic of humanitarian medicine. Humanitarian medicine was conceptualized by Sorensen as a realm of functional limitations where standardized guidelines (based on those created by MSF) were to be relied upon in determining care instead of more individualized diagnostic interactions between doctors and patients, which would have been more time-intensive and required a diversity of specialized medical expertise. As Nicolas Dodier also observed in the practices of occupational medicine for manual labourers in France, medical interactions were to be governed by a set of standardized practices and drugs, which responded to an expected list of symptoms rather than a more ‘clinical’ diagnostic approach.^141^ This emphasis on drug supply at the heart of medical intervention has been described as pharmaceuticalization and it is indeed shifting the politics of care to that of a pharmaceutical marketplace. The kits have, in this respect, enabled the shift of focus in humanitarian medicine from the urgency of care and the volume of deployment of qualified workforce to a focus on access to drugs and tightly regulated clinical practice.^142^ In short, the ‘humanitarian emergency’ has been delineated as a space that demands a focus on rapidity to the detriment of what is now termed patient- or person-centred care.^143^ The practicality of streamlined care in emergency contexts was undeniable, yet in deciding exactly what clinical guidelines and drugs would govern patient outcomes, the mostly European medical professionals who contributed to these discussions moulded a humanitarian medicine primarily influenced by the ‘supply’ side of the relief interaction in terms of resource and logistical considerations and biopower considerations of quality control and disciplined processes.^144^

Equally, the promoters of the kits may have invested them with too much significance at the time and since. In reality, it makes more sense to describe the kit as the centrepiece of a discursive exchange between humanitarian norm shapers rather than an ‘immutable object’. Nevertheless, the kits represented the engagement of an aid sector concerned with its own rapid growth. The mobility the kit embodied was aligned, to an extent, with the new supply-chain theories of just-in-time stockkeeping. However, humanitarian logistics was not established with a view to smooth frictions, but in the expectation of heading directly towards likely disruption and accidents. Transcending trade zones and often creating rules of exceptions for themselves, the UN and INGOs’ transiting of drugs, including psychoactive substances or low-cost alternatives, was in many ways the expression of a free trade, *sans-frontiérisme*, which matched liberal discourses of the period.^145^ As actors in the humanitarian marketplace, the large UN agencies such as UNICEF and UNHCR and the larger INGOS developed authoritative and, through field testing, proprietary processes focused on standardization. The creation and diffusion of products like the Emergency Health Kit emerged from the competitive environment of the humanitarian sector but soon reflected the entrenched positions of its key market leaders. Oxfam provided its WATSAN (Water and Sanitation) kits, while MSF and UNICEF developed expertise which fed into WHO guidelines and enabled them to become market-leading suppliers.

The ‘marketplace’ in which kits were deployed was not only skewed by the expectation of urgency and disruption; it was also defined by its contribution to a supply-led economic exchange governed by the need to control people, products, and markets.^146^ While the drive to reduce costs and divergences in medical practice in clinical care was clearly assumed, its authoritarianism was presented as a necessity under the guise of adapting to austerity and developing an adaptable workforce. Going against the grain of medical professionalization, it proposed de-specialization as an ideal. As a liberal ideal (if not a neoliberal one), the constraints the kits represented would, in the 1990s, be construed as essential building blocks of accountability. The possible improvement in quality and efficiency of care, and the greater mobility of drugs and equipment for essential medical care, resulted in higher accountability of humanitarian relief to aid recipients, echoed the increased basic accountability in stocktaking, and related accounting for medical products as depreciating resources.

By focusing on the material culture of aid and its ideological baggage, we can also see how the development of the kits showed the deep-seated contestation of what emergency relief represented and how its very existence challenged more aspirational development ideals. As the token expression of emergency solidarity in the 1980s, kits could be perceived as its limit, even though they were never really intended as anything more than a framing device to kick-start a humanitarian response. The diversification of kits, and the increasing complexity of drug lists since the 1990s, has reduced the centrality of the standardized Emergency Health Kit. Nevertheless, the normative discussions on the kit reveal how localized spaces played a strong role in reproducing distinctions between ‘international’ and ‘local’ staff in aid work, while the ‘reification of a mobile space of response’ implied by the kit remains a key feature of the contemporary material culture of aid.^147^ Alongside the kits emerged the figure of the humanitarian logistician and pharmacist whose role was to enable the deployment of materials, including kits, and to ensure the smooth rotation of medical teams. If those medical teams were forced into accepting de-specialization as part of their engagement with austere environments, logisticians, in contrast, were expected to develop complex professional skills meeting the varied demands of their environment. The chains of command and supply the kits represented implied a stable medical and scientific environment which may, through the inertia of painful compromises, have turned into innovation traps.

## Footnotes

1 Tom Scott-Smith, ‘Infrastructural Immobility: Movement and Material Culture in the Calais Jungle’, *Incarceration* 3, no. 1 (2022): 1–13.

2 Peter Redfield, ‘Vital Mobility and the Humanitarian Kit’, in *Biosecurity Interventions: Global Health and Security in Question*, ed. Andrew Lakoff and Stephen J. Collier (Columbia University Press, 2008), 147–72.

3 Tom Scott-Smith, ‘The Fetishism of Humanitarian Objects and the Management of Malnutrition in Emergencies’, *Third World Quarterly* 34, no. 5 (2013): 913–28; Tom Scott-Smith, ‘Control and Biopower in Contemporary Humanitarian Aid: The Case of Supplementary Feeding’, *Journal of Refugee Studies* 28, no. 1 (2015): 21–37; Kristin Bergtora Sandvik et al., ‘Humanitarian Technology: A Critical Research Agenda’, *International Review of the Red Cross* 96, no. 893 (2014): 219–42; Kristin Bergtora Sandvik, *Humanitarian Extractivism: The Digital Transformation of Aid* (Manchester University Press, 2023); Joël Glasman, *Humanitarianism and the Quantification of Human Needs: Minimal Humanity* (Routledge, 2019).

4 UNICEF Supply Division, ‘Liam Neeson at the World’s Largest Humanitarian Warehouse’, accessed 13 September 2024, https://www.youtube.com/watch?v=wWq3RjBhXlg. For more on the centrality of kits to humanitarian promotion, see Heike Wieters, *The NGO Care and Food Aid from America 1945*–*80:* “*Showered with Kindness*”*?* (Manchester University Press, 2017).

5 Tom Scott-Smith, ‘Sticky Technologies: Plumpy’nut®, Emergency Feeding and the Viscosity of Humanitarian Design’, *Social Studies of Science* 48, no. 1 (2018): 3–24; Joël Glasman, ‘Measuring Malnutrition: The History of the MUAC Tape and the Commensurability of Human Needs’, *Humanity* 9, no. 1 (2018): 19–44.

6 Bruno Latour, ‘When Things Strike Back: A Possible Contribution of “Science Studies” to the Social Sciences’, *British Journal of Sociology* 51, no. 1 (2000): 107–23. For a definition of black-boxing, see Latour, *Pandora*’*s Hope: Essays on the Reality of Science Studies* (Harvard University Press, 1999), 272.

7 *WHO Emergency Health Kit* (WHO, 1984), accessed 13 September 2024, https://iris.who.int/handle/10665/44255?search-result=true&query=emergency+health+kit+1984&scope=&rpp=10&sort_by=score&order=desc.

8 UNICEF played a major role in supplying the kits through UNIPAC in Copenhagen, but the UNICEF Archives in New York are closed for the foreseeable future. The International Committee of the Red Cross also played a role in adapting the New Emergency Health Kit, but most of its archival collections have a fifty-year embargo policy. UNHCR Archives in Geneva currently have a wait time of more than a year to receive a research appointment (and very limited online finding aides). We therefore relied on personal papers of experts with ties to ICRC and UNICEF, including Ron Ockwell and Rudi Coninx, and on UNHCR, ICRC, and UNICEF documents retained at the WHO Archives in Geneva.

9 João Guilherme Biehl, ‘Pharmaceuticalization: AIDS Treatment and Global Health Politics’, *Anthropological Quarterly* 80, no. 4 (2007): 1083–126.

10 For MSF it was the appearance of fake meningitis vaccines in 1995 in Niger and antibiotics, with up to 50% of the latter failing conformity tests in the 1990s. Papiers Jacques Pinel, box 4 médicaments, MSF. See also Christopher Sirrs, ‘Fluid Fakes, Contested Counterfeits: The World Health Organization’s Engagement with Fake Drugs, 1948–2017’, *Medical Anthropology Theory* 10, no. 3 (2023): 1–29.

11 Jeremy Greene, ‘Making Medicines Essential’: The Emergent Centrality of Pharmaceuticals in Global Health’, *BioSocieties* 6 (2011): 10–33.

12 Maurice Cassier and Carine Baxerres, ‘Conclusion: Rationalizing Drug Markets in the Global South: Re-Making Medicines Essential’, in *Understanding Drugs Markets*, ed. Maurice Cassier and Carine Baxerres (Routledge, 2022), 274–91.

13 Mohammad Moshtari et al., ‘Procurement in Humanitarian Organizations: Body of Knowledge and Practitioner’s Challenges’, *International Journal of Production Economics* 233 (2021): 108017.

14 Cassier and Baxerres, ‘Rationalizing Drug Markets in the Global South’, 274.

15 Emanuel Adler and Steven Bernstein, ‘Knowledge in Power: The Epistemic Construction of Global Governance’, in *Power in Global Governance*, ed. Michael Barnett and Raymond Duvall (Cambridge University Press, 2004), 294–318.

16 Kevin O’Sullivan, *The NGO Moment: The Globalisation of Compassion from Biafra to Live Aid* (Cambridge University Press, 2021), 34–55; Bertrand Taithe, ‘The Cradle of the New Humanitarian System? International Work and European Volunteers at the Cambodian Border Camps, 1979–1993’, *Contemporary European History* 25, no. 2 (2016): 335–58.

17 On the primary health care approach of the Somali government, see Nancy Godfrey and Husein Mursal, ‘International Aid and National Health Policies for Refugees: Lessons from Somalia’, *Journal of Refugee Studies* 3, no. 2 (1990): 110–13; oral history interview with Abdi Shire Jama, 25 October 2024; oral history interview with Mohamed Elmi, 16 December 2024; oral history interview with Mohamed Jama, 26 September 2024. On the founding principles and national leadership for the Refugee Health Unit, see ‘The Somalia Refugee Emergency: International Assistance in the Long Term’ [1980], folder 2, box PRG/5/5/60, Oxfam Archive, Oxford (hereafter, Oxfam); Tim Brierly, re: SOM. 10 – Basic Health Services, 10 September 1978, folder Somalia 10 Vol. 3, box PRF 3703, Oxfam; RHU Newsletter, Issue no. 10, March 1982, RHU Dropbox (private archive compiled by Beverley Snell and Maria Therese Lenz-Dualeh); Fourth Semi-Annual Health Unit Workshop Summary of Proceedings, box Somalie 1981–1982 (UNHCR), Médecins Sans Frontières Archives, Paris (hereafter, MSF).

18 Such as the Mid-Upper Arm Circumference or MUAC tape, and the Ready to Use Therapeutic Food Plumpy’Nut. See Glasman, ‘Measuring Malnutrition’; Scott-Smith, ‘Sticky Technologies’.

19 Jean Pierre Hiegel, ‘The ICRC and Traditional Khmer Medicine’, *International Review of the Red Cross (1961*–*1997)* 21, no. 224 (1981): 251–62; Cora Neumann and Gerard Bodeker, ‘Humanitarian Responses to Traditional Medicine for Refugee Care’, in *Traditional, Complementary and Alternative Medicine: Policy and Public Health Perspectives*, ed. Gerard Bodeker, Gemma Burford, and Fredi Kronenberg (Imperial College Press, 2007), 185–204. For more on the Christian Medical Commission and traditional medicine in Africa in the 1970s–1980s, see Traditional Healing folders, box 4215.2.13, Christian Medical Commission collection, World Council of Churches Archives, Geneva (hereafter, CMC).

20 Kevin O’Sullivan, ‘Aidland in South Asia: Humanitarian Crisis and the Contours of the Global Aid Industry in the Long 1970s’, *European Review of History* 29, no. 3 (2022): 499–519.

21 See Stephen J. Collier and Andrew Lackoff, ‘Vital Systems Security: Reflexive Biopolitics and the Government of Emergency’, *Theory, Culture & Society* 32, no. 2 (2015): 19–51; Peter Adey, ‘Emergency Mobilities’, *Mobilities* 11, no. 1 (2016): 32–48; Heidi Kaspar, Margaret Walton-Roberts, and Audrey Bochaton, ‘Therapeutic Mobilities’, *Mobilities* 14, no. 1 (2019): 1–19. With many thanks to Stephanie Sodero for her guidance.

22 Concerns for logistics first emerged in relation to global war efforts, and the globalization of supply chains found its first expression in Second World War supply lines. Richard M. Leighton and Robert W. Coakley, *Global Logistics and Strategy, 1940*–*1943*, vol. 1 (Office of the Chief of Military History, Dept. of the Army, 1955). On supply chain, see Huiping Ding, ‘History Issues and Future Scope of Logistics and Supply Chain’, *Journal of Enterprise and Business Intelligence* 3, no. 2 (2023): 95–105; Deborah Cowen, *Deadly Life of Logistics: Mapping Violence in Global Trade* (University of Minnesota Press, 2014), especially ch. 2. On shifting (governmental) conceptions of spatiality and responding to emergencies within a ‘vital systems security’ framework, see Collier and Lackoff, ‘Vital Systems Security’.

23 This was the case in Somalia. Interview with Beverley Snell, 12 April 2024.

24 While international organizations were often spaces for contestation and Global South mobilization in the post-colonial period, in this sense the WHO and NGOs played a role in preserving a hierarchical global economy in health goods. See ‘Special Issue: Towards a Global History of International Organizations and Decolonization’, *Journal of Global History* 17, no. 2 (2022). Within this Special Issue, see Bogdan C. Iacob, ‘Malariology and Decolonization: Eastern European Experts from the League of Nations to the World Health Organization’, 233–53.

25 Stephen Hopgood, ‘Saying “No” to Wal-Mart? Money and Morality in Professional Humanitarianism’, in *Humanitarianism in Question*, ed. Michael N. Barnett and Thomas G. Weiss (Cornell University Press, 2018), 98–123.

26 Matthias Middell and Katja Naumann, ‘Global History and the Spatial Turn: From the Impact of Area Studies to the Study of Critical Junctures of Globalization’, *Journal of Global History* 5, no. 1 (2010): 149–70, 151–2.

27 Peter Walker and Susan Purdin, ‘Birthing Sphere’, *Disasters* 28 no. 2 (2004):100–11; Margie Buchanan-Smith, *How the Sphere Project Came into Being: A Case Study of Policy-Making in the Humanitarian Aid Sector and the Relative Influence of Research* (Overseas Development Institute, 2003). This perspective was contested in Jacqui Tong, ‘Questionable Accountability: MSF and Sphere in 2003’, *Disasters* 28, no. 2 (2004): 176–89; Charlotte Dufouret et al., ‘Rights, Standards and Quality in a Complex Humanitarian Space: Is Sphere the Right Tool?’ *Disasters* 28, no. 2 (2004): 124–41; Fabien Dubuet, ‘What Is Humanitarian Accountability?’, *ICRC Forum* (April 2002), accessed 4 September 2024, https://www.msf.fr/sites/default/files/2002-04-01-Dubuet.pdf.

28 On the role of material culture in making the sites of international aid into ‘liminal spaces’, see Lisa Smirl, *Spaces of Aid: How Cars, Compounds and Hotels Shape Humanitarianism* (Zed Books, 2015); Smirl, ‘Building the Other, Constructing Ourselves: Spatial Dimensions of International Humanitarian Response’, *International Political Sociology* 2 (2008), 236–53; David Mosse, *Adventures in Aidland: The Anthropology of Professionals in International Development* (Berghahn Books, 2011).

29 Peter Redfield (2008), quoted in Adey, ‘Emergency Mobilities’.

30 Interview with Jacques Pinel, 22 June 2018; Interview with Jean Rigal, 6 October 2023.

31 ’Water treatment’ correspondence, PRG/5/3/7/3, Oxfam. Nutritional guidelines originated in the 1974 Ethiopian famine. See Tim Lusty and Pat Diskett, ‘Selective Feeding Programmes’, Oxfam Practical Guide No. 1 (Oxfam, 1984).

32 ’Christian Medical Commission Programme on Getting Essential Drugs to the People through Cooperative Pharmaceutical Services, Southern Sudan, 24 Sept–15 Oct 1982’, box 4215.2.9, folder 1, CMC.

33 Richard Laing et al., ‘25 Years of the WHO Essential Medicines Lists: Progress and Challenges’, *Lancet* 361 no. 9370 (2003): 1723–9. The list is reviewed every two years by WHO and by 2007 it had grown to over 300 drugs. See Marcus M. Reidenberg, ‘World Health Organization Program for the Selection and Use of Essential Medicines’, *Clinical Pharmacology and Therapeutics* 81, no. 4 (April 2007): 603–6; Bernadette Cappello et al., ‘The “Square Box”: Therapeutic Equivalence as a Foundation of the WHO Model List of Essential Medicines’, *Frontiers in Pharmacology* 11 (2020): Article 578000.

34 ’WHO Emergency Health Kit: Standard Drugs and Clinic Equipment for 3 Months’ (Geneva: World Health Organization, 1984). See also the 1987 draft of the Emergency Health Kit in the Rudi Coninx papers, Humanitarian Archive, John Rylands Library, Manchester (hereafter, HAJRL).

35 Vincent Fauveau, ‘Pour une médecine différente en milieu defavorisé’, Papiers Jacques Pinel box 2, MSF.

36 See Anne Vallaeys, *Médecins Sans Frontières: La biographie* (Fayard,1992), 324–5; Roger Glass et al., ‘Rapid Assessment of Health Status and Preventive-Medicine Needs of Newly Arrived Kampuchean Refugees, Sa Kaeo, Thailand’, *Lancet* 315, no. 8173 (1980): 868–872; Christel Thibault, *L*’*Archipel des camps: L*’*exemple cambodgien* (Presses Universitaires de France, 2008), 36–9.

37 William Shawcross, *The Quality of Mercy: Cambodia, Holocaust, and Modern Conscience* (Simon and Schuster, 1984), 175–8.

38 Interview with Vincent Fauveau, 8 December 2023. See also Claire Escoffier-Fauveau, *L*’*Enfant sans frontières* (Éditions des Quatres Seigneurs, 2022), 109.

39 Taithe, ‘The Cradle of the New Humanitarian System?’, 337.

40 Bertrand Taithe, ‘Between the Border and a Hard Place: Negotiating Protection and Humanitarian Aid after the Genocide in Cambodia, 1979–1999’, in *Humanitarianism and Human Rights*, ed. Michael Barnett (Cambridge University Press, 2020), 219–34. MSF also had a small presence in camps at the Thai–Cambodian border since 1976, before the large-scale crisis in 1979. See Vallaeys, *Médecins Sans Frontières*, 234–7.

41 Taithe, ‘The Cradle of the New Humanitarian System?’, 350. See J. David Kinzie, ‘Therapeutic Approaches to Traumatized Cambodian Refugees’, *Journal of Traumatic Stress* 2, no. 1 (1989): 75–91.

42 Soren C. Sorensen, Travel report on visit to camps for displaced populations in Thailand (11 May 1987), E17-374-2, WHO.

43 Our reflections on Somalia and the Refugee Health Unit that oversaw refugee relief there in the 1980s are based on consultation of documents in the MSF, Oxfam, WHO, and Christian Medical Commission archives, along with oral history interviews conducted with fourteen expatriate and Somali practitioners who were involved in the programme, and the private archives of the Refugee Health Unit kept by some of these people and shared with us for this research. See, for example, Refugee Health Unit, *Guidelines for Health Care in Refugee Camps of the Somali Democratic Republic* (Somali Ministry of Health, 1981); interview with Ahamed Magan, 19 July 2024; ‘Rapport Final Mission MSF Somalie Janvier 1983’, Somalie 1981–1985, MSF.

44 10 June 1986 report on Somalia sent by Abdilahi Hassan Farah and primary health-care training officer Yusuf Mohamed Abdilahi to S. C. Sorensen, and reply from S. C. Sorensen to Beverley Snell on 7 January 1987, E-17-180 SOM, Jacket 3, WHO.

45 CEE Thaïlande, Thaïlande 1983, MSF; Vallaeys, *Médecins Sans Frontières*, 307–60.

46 Guidelines written by Vincent Fauveau and Jean Rigal eventually became formalized MSF clinical guides that fed into WHO Emergency Health Kit. Interview with Vincent Fauveau, 8 December 2023. Interview with Jean Rigal, 6 October 2023. Jacques Pinel to S. C. Sorensen, 18 December 1986, E17-374-2, WHO.

47 Letter from F. S. Antezana (Action Programme on Essential Drugs) to Stephanie Simmonds, 30 August 1990, E17-374-2, WHO.

48 Interview with Martijn Blansjaar, 20 February 2024. Blansjaar described how the absence of drugs for chronic diseases was only perceived as a problem when kits were used in Europe in the 1990s and found to be inadequate for the emergency needs of Europeans with chronic diseases.

49 Letter from Antezana to Simmonds, 30 August 1990.

50 MSF documents (‘WHO/UNHCR Emergency Health Kit: Bases de discussion pour sa révision’), 12/1986, E17-374-2, WHO.

51 Emergency Health Kit (July 1987), 2, Rudi Coninx papers, HAJRL.

52 Ibid.

53 Letter from Antezana to Simmonds, 30 August 1990; Hans Hogerzeil, Health Kit, 1989 (introductory document), E17-374-2, WHO.

54 S. C. Sorensen to Jacques Pinel, 8 December 1986 and Jacques Pinel to S. C. Sorensen, 18 December 1986, E17-374-2, WHO.

55 ’WHO Emergency Health Kit … Project Proposal for Evaluation and Revision’ (November 1986), E17-374-2, WHO.

56 Later renamed Interagency Emergency Health Kit, revised in 2011 to develop mental health and child needs and reviewed in relation to malaria in 2015 (see Figure 1). Health Emergency Programme, *The Interagency Emergency Health Kit, Medicine and Medical Devices for 10*,*000 People for Approximately Three Months* (WHO, 2017), 7.

57 Aspect de la logistique, 10 January 1992, box 4 ‘Médicaments’, Papiers Jacques Pinel, MSF.

58 Slim Slama et al., ‘The Development of the Noncommunicable Diseases Emergency Health Kit’, *Eastern Mediterranean Health Journal* 24, no. 1 (2018): 92–8.

59 By 2017, the kit including the malaria unit and associated supplementary modules weighed 1150 kg. *The Interagency Emergency Health Kit*, 10.

60 See https://www.who.int/emergencies/emergency-health-kits, accessed 19 February 2024.

61 This pilot kit included several herbal medicines and traditional Chinese medicine. Juan Alfonso Leonardia and Paul Andrew G Zambrano, ‘Development of a Traditional Medicine Disaster Unit’, *Southeast Asian Journal of Tropical Medicine and Public Health* 40, no.1 (2009): 96-105.

62 Hans Hogerzeil, New Emergency Health Kit, 1989 (introductory document), E17-374-2, WHO.

63 Letter from Jacques Pinel to S. C. Sorensen, 3 December 1986, E17-374-2, WHO.

64 O’Sullivan, ‘Aidland in South Asia’.

65 Interview with Vincent Fauveau; Escoffier-Fauveau, *L*’*Enfant sans frontières*.

66 Interview with Vincent Fauveau.

67 B. Silve, ‘Health Logistics Is a Profession: Improving the Performance of Health in Developing Countries’, *Field Actions Science Reports. The Journal of Field Actions* 1, no. 2 (2008): 19–25, 20.

68 Interview with Vincent Fauveau.

69 First draft of Emergency Manual, April 1983, Ron Ockwell papers, HAJRL.

70 Tim Lusty and Pat Diskett, *Supplementary Feeding Programmes* (Oxfam, 1984).

71 Paul Rabinow and Nikolas Rose, ‘Biopower Today’, *BioSocieties* 1, no. 2 (2006): 195–217.

72 Maria Cullen, ‘NGOs as National Political Actors during the Cold War: A Comparison of Médecins Sans Frontières and Oxfam’s Humanitarian Programmes in the Global South, 1979–1988’ (PhD diss., University of Galway, 2023), chs. 2 and 4.

73 Final draft of UNICEF handbook 1985, Ron Ockwell papers, HAJRL.

74 Emily Baughan, S*aving the Children: Humanitarianism, Internationalism, and Empire* (University of California Press, 2022); Bertrand Taithe, ‘Reinventing (French) Universalism: Religion, Humanitarianism and the “French Doctors”‘, *Modern & Contemporary France* 12, no. 2 (2004): 147–58; Agnieszka Sobocinska, *Saving the World?: Western Volunteers and the Rise of the Humanitarian-Development Complex* (Cambridge University Press, 2021); O’Sullivan, *The NGO Moment*.

75 Pierre Hiegel, ‘Collaboration with Traditional Healers: Experience in Refugees’ Mental Care’, *International Journal of Mental Health* 12, no. 3 (1983): 30–43; ‘La Médecine traditionelle Khmère’ [1981], Thaïlande 1983, MSF.

76 UNBRO, Health Statistics for Thai–Kampuchean Border Camps, Annual Report 1988 – Summary, Coninx papers, HAJRL.

77 Randall Packard, *A History of Global Health: Interventions into the Lives of Other Peoples* (Johns Hopkins University Press, 2016), 227–66. Also see World Health Organization, *The Third Ten Years of the World Health Organization* (WHO, 2008); Marcos Cueto, ‘The Origins of Primary Health Care and Selective Primary Health Care’, *American Journal of Public Health* 94, no. 11 (2004): 1864–74.

78 George Weisz and Noémi Tousignant, ‘International Health Research and the Emergence of Global Health in the Late Twentieth Century’, *Bulletin of the History of Medicine* 93, no. 3 (2019): 365–400. See also Martin Gorsky and Christopher Sirrs, ‘World Health by Place: The Politics of International Health System Metrics, 1924–c. 2010’, *Journal of Global History* 12, no. 3 (2017): 361–85.

79 Final draft of original UNICEF handbook 1985 (see ‘Internal Management Actions’, 56), Ron Ockwell papers, HAJRL.

80 Ibid.

81 S. C. Sorensen to Dr Per Juul (Farmaceutisk Hojsole), 8 December 1986, E17-374-2, WHO.

82 Eleuther Tarimo and E. G. Webster, ‘Primary Health Care Concepts and Challenges in a Changing World: Alma-Ata Revisited’, ARA paper 7 (WHO, 1997), 5–7.

83 Tom Scott-Smith, *On an Empty Stomach: Two Hundred Years of Hunger Relief* (Cornell, 2020), 139.

84 Sorensen, Travel report on visit to camps for displaced populations in Thailand (11 May 1987).

85 ’WHO Emergency Health Kit’ (November 1986), E17-374-2, WHO.

86 Courtland Robinson, ‘Refugee Warriors at the Thai–Cambodian border’, *Refugee Studies Quarterly* 19, no. 1 (2000): 23–37; Norah Niland, ‘The Politics of Suffering: the Thai–Cambodian Border, a Case Study on the Use and Abuse of Humanitarian Assistance’ (M.Phil diss., Trinity College Dublin, 1991).

87 See Fiona Terry, *Condemned to Repeat? The Paradox of Humanitarian Action* (Cornell University Press, 2002).

88 13 July letter from WFP/UNBRO official to Voluntary Agency Directors, Thaïlande 1983, MSF.

89 Report compiled by Gilles Germain, Ta Phraya-Lyon, 15 Août–15 Octobre 1984 (first page of report not visible), Thaïlande 1984: courriers/comptes, rapports diverses, MSF.

90 Ibid.

91 Rudi Coninx Dong Ruk report, December 1984, Thaïlande 1984: courriers/comptes, rapports diverses, MSF.

92 MSF documents, ‘WHO/UNHCR Emergency Health Kit: Bases de discussion pour sa révision’, 12/1986, ‘Protocole d’enquête sur la consommation médicamenteuse’, E17-374-2, WHO.

93 Interview with Jean Rigal, 6 October 2023.

94 Interview with Martijn Blansjaar, 20 February 2024.

95 Interview with Rudi Coninx, 20 March 2024.

96 Renée C. Fox, *Doctors without Borders: Humanitarian Quests, Impossible Dreams of Médecins Sans Frontières* (John Hopkins University Press, 2014).

97 Papiers Jacques Pinel, box Kit 1 débuts des années 80, MSF.

98 It began in the 1940s at WHO, but by 1975 acquired new momentum, with the non-aligned movement adopting a resolution (1976) pushing developing countries to cooperate with international organizations on pharmaceutical policy for more equitable production/procurement/distribution of drugs. Mahler spoke on essential drugs issue in a World Health Assembly 1975 address and asked the pharmaceuticals division at WHO to form an expert committee, which would establish the ‘essential drugs philosophy’ and create the list of essential drugs, as well as provide technical advice on its roll-out. See Greene, ‘Making Medicines Essential’.

99 Papiers Jacques Pinel, Listes – WHO, The Emergency Health Kits, explanation, 1, MSF.

100 Papiers Jacques Pinel, Listes – liste des médicaments et matériels, February 1986, MSF.

101 MSF documents (‘WHO/UNHCR Emergency Health Kit: Bases de discussion pour sa revision’).

102 Manjit Kaur and Sarah Hall, *ECHO: Medical Supplies and Equipment for Primary Health Care, a Practical Resource for Procurement and Management* (ECHO International, 2001).

103 ECHO: Basic Equipment Kit for the Rural Health Centre, Débuts des kits, Papiers Jacques Pinel, MSF.

104 Débuts des kits, MSF Belgique, 1984, Papiers Jacques Pinel, MSF.

105 S. C. Sorensen to Peter Bulling (Danish Red Cross), 8 December 1986, E17-374-2, WHO; UNICEF Book B 5, f/ provision of essential drugs, Ockwell papers, HAJRL.

106 MSF documents (‘WHO/UNHCR Emergency Health Kit: Bases de discussion pour sa révision’).

107 ’Comments on Project Proposal for WHO Emergency Health Kit’, EMRO/ PTS, 1 December 1986, E17-374-2, WHO.

108 Udani Samarasekera, ‘Unitaid at 15’, *Lancet* 398, no. 10305 (2021): 1032–3.

109 Claudine Vidal and Jacques Pinel, ‘MSF “Satellites”: A Strategy Underlying Different Medical Practices’, in *Medical Innovations in Humanitarian Situations*, ed. Jean-Herve Bradol and Claudine Vidal (MSF 2011), 22–39.

110 Lin Zhang et al., ‘Materials Procurement and Reserves Policies for Humanitarian Logistics with Recycling and Replenishment Mechanisms’, *Computers & Industrial Engineering* 127 (2019): 709–21.

111 D. A. Richardson, S. de Leeuw, and W. Dullaert, ‘Factors Affecting Global Inventory Prepositioning Locations in Humanitarian Operations – a Delphi Study’, *Journal of Business Logistics*, 37 no. 1 (2016): 59–74.

112 The debates on fiduciary responsibilities in humanitarianism reflected contested political legitimacy of humanitarian actors, with UN agencies in particular exercising fiduciary responsibility to act in the best interest of those depending on their trusteeship. Allen Buchanan, ‘The Internal Legitimacy of Humanitarian Intervention’, *Journal of Political Philosophy* 7, no. 1 (1999): 71–87, 76.

113 Luk N. Van Wassenhove, ‘Humanitarian Aid Logistics: Supply Chain Management in High Gear’, *Journal of the Operational Research Society* 57, no. 5 (2006): 475–89; Yousuf Akbari Shahrestanaki et al., ‘Organization and Preparedness of Emergency and Disaster Medical Response Teams: Obstacles and Challenges’, *Health in Emergencies and Disasters Quarterly* 5, no. 4 (2020): 169–82.

114 A. Tcheknavorian, ‘The Pharmaceutical Industry: The Problems and the Opportunities’, United Nations Industrial Development Organization, Vienna, 1981, box 4215.2.10, CMC.

115 Rägo Lembit and Budiono Santoso, ‘Drug Regulation: History, Present and Future’, *Drug Benefits and Risks: International Textbook of Clinical Pharmacology* 2 (2008): 65–77. The European Economic Committee tightened regulations between 1975 and 1987, before the establishment of the European Medicines Evaluation Agency in 1993.

116 John Abraham, ‘Pharmaceuticals, the State and the Global Harmonisation Process’, *Australian Health Review* 28, no. 2 (2004): 150–60. For a full timeline of international quality assurance processes, see ‘Norms and Standards: 70 Years of WHO Standards on Medicines Quality’, *WHO Drug Information* 31, no.1 (2017): 15–26, accessed on 23 August 2024, https://iris.who.int/bitstream/handle/10665/330978/DI311-15-26-eng.pdf.

117 Aisling Leow, James Hu, and Tom Hird, ‘An Overview of WHO Prequalification’, Rethink Priorities (2023), accessed on 17 September 2024, https://rethinkpriorities.org/publications/an-overview-of-who-prequalification-process-usage-and-potential-improvements.

118 Ellen F. M. ‘t Hoen et al., ‘A Quiet Revolution in Global Public Health: The World Health Organization’s Prequalification of Medicines Programme’, *Journal of Public Health Policy* 35 (2014): 137–61.

119 According to the WHO’s initial benchmarking of national drug regulatory systems, within its regulatory system strengthening programme in 1997. This system later evolved into a streamlined Global Benchmarking Tool, which was first released in 2013. Alireza Khadem Broojerdi et al., ‘The World Health Organization Global Benchmarking Tool: An Instrument to Strengthen Medical Products Regulation and Promote Universal Health Coverage’, *Frontiers in Medicine* 7, no. 457 (2020): 1–7.

120 ’t Hoen, et al., ‘A Quiet Revolution’, 140.

121 Papiers Jacques Pinel, box 4, logistique, MSF. This is an ongoing issue; see Marie Elske Gispen, *Human Rights and Drug Control: Access to Controlled Essential Medicines in Resource-Constrained Countries* (Cambridge University Press, 2017).

122 Interview with Beverley Snell, 12 April 2024.

123 S. C. Sorensen report, 26 March 1987, on visit to Regional Office for South-East Asia, New Delhi, India, E17-374-2, WHO.

124 See Erin R. Graham, ‘International Organizations as Collective Agents: Fragmentation and the Limits of Principal Control at the World Health Organization’, *European Journal of International Relations* 20, no. 2 (2014): 366–90; Marcos Cueto, Theodore Brown, and Elizabeth Fee, *The World Health Organization: A History* (Cambridge University Press, 2019).

125 Letter from G. Szalay (Purchasing Officer, org unclear) to S. C. Sorensen, 6 April 1987, E17-374-2, WHO.

126 Ibid.

127 See Monica Prasad, *The Politics of Free Markets: The Rise of Neoliberal Policies in Britain, France, Germany, and the United States* (University of Chicago Press, 2006).

128 The WHO Archives have numerous documents describing the medical marketplace of the 1970s–1980s and the discussions with industry actors about the concept of essential drugs. See, for instance, Meetings of the International Federation of Pharmaceutical Manufacturers Associations, Jackets 2–3, P 5/86/13, WHO; Relations with the International Federation of Pharmaceutical Manufacturers Associations, Jackets 2–3, Folio 31, P 5/348/4, WHO.

129 Emergency Health Kit 1987, Rudi Coninx papers, AHJR. See the failed attempt by MSF to organize a Nairobi hub with the Centrale Humanitaire Médico-pharmaceutique in the 1990s, Papiers Jacques Pinel box 4, CHMP, MSF.

130 S. C. Sorensen to J. Tulloch, CDD, 9 December 1986, E17-374-2, WHO.

131 Sorensen to Juul, 8 December 1986.

132 Ibid.

133 Sorensen to Snell, 7 January 1987.

134 *WHO Bacterial Priority Pathogens List, 2024: Bacterial Pathogens of Public Health Importance to Guide Research, Development and Strategies to Prevent and Control Antimicrobial Resistance* (WHO, 2024).

135 *WHO Bacterial Priority Pathogens List*, x, 1–2; L. P. Haraoui et al., ‘Armed Conflicts and Antimicrobial Resistance: A Deadly Convergence’, *AMR Control* 5 (2019): 69–73; Jacob Goldberg, Angela Uyen-Cateriano, and Gijs Van Gassen, ‘Making Matters Worse: Antimicrobial Resistance in Conflict Settings’, Revive: Advancing Antimicrobial R&D (23 June 2022), accessed on 22 July 2024, https://revive.gardp.org/making-matters-worse-antimicrobial-resistance-in-conflict-settings/.

136 S. C. Sorensen to D. B. Doberstyn, ‘Emergency Health Kit’, 4 December 1986, E17-374-2, WHO.

137 S.C. Sorensen to Jacques Pinel, 7 January 1987, E17-374-2, WHO.

138 In 1994, chloroquine remained the WHO’s favoured option, while experts acknowledged the growing problem of falciparum malaria. See David Alnwick (Project Manager, RBM) to Hans Hogerzeil (Coordinator, PAR), ‘Update of the new Emergency Health Kit 1998’, 22 November 2002, E19-374-16, WHO; José A. Nájera, ‘Malaria Control among Refugees and Displaced Populations’ (WHO Division of Control of Tropical Diseases Malaria Unit, 1996).

139 Suna Balkan and Jean-François Corty, ‘Malaria: Introducing ACT from Asia to Africa’, in *Medical Innovations*, ed. Bradol and Vidal; CDC, ‘Choosing a Drug to Prevent Malaria’, accessed on 22 July 2024, https://www.cdc.gov/malaria/hcp/drug-malaria/index.html.

140 S. C. Sorensen to Olavi Elo, 7 January 1987, E17-374-2, WHO.

141 Nicolas Dodier, ‘Clinical Practice and Procedures in Occupational Medicine: A Study of the Framing of Individuals’, in *Differences in Medicine: Unraveling Practices, Techniques, and Bodies*, ed. Marc Berg and Annemarie Mol (Duke University Press, 1998), 53–85.

142 This is a development which was directly related to the debates on access to drugs prevailing in the 1990s response to AIDS. Biehl, ‘Pharmaceuticalization: AIDS Treatment and Global Health Politics’.

143 Angela Coulter and John Oldham, ‘Person-Centred Care: What Is It and How Do We Get There?’, *Future Hospital Journal* 3, no. 2 (2016): 114–16.

144 Norbert Götz, Georgina Brewis, and Steffen Werther, *Humanitarianism in the Modern World: The Moral Economy of Famine Relief* (Cambridge University Press, 2020).

145 Eleanor Davey, *Idealism beyond Borders: The French Revolutionary Left and the Rise of Humanitarianism, 1954*–*1988* (Cambridge University Press, 2015).

146 On the recent use of E. P. Thompson’s theory of moral economy in analyses of humanitarian relief, see Götz, Brewis, and Werther, *Humanitarianism in the Modern World*.

147 Smirl, *Spaces of Aid*, 3; Smirl, ‘Building the Other, Constructing Ourselves’, 243.

